# MMIR: an open-source software for the registration of multimodal histological images

**DOI:** 10.1186/s12911-024-02424-3

**Published:** 2024-03-05

**Authors:** Rodrigo Escobar Díaz Guerrero, José Luis Oliveira, Juergen Popp, Thomas Bocklitz

**Affiliations:** 1 BMD Software, PCI - Creative Science Park, 3830-352, Ilhavo, Portugal; 2 DETI/IEETA, University of Aveiro, 3810-193 Aveiro, Portugal; 3 Leibniz Institute of Photonic Technology Jena, Member of Leibniz research alliance ‘Health technologies’, Albert-Einstein-Straße 9, 07745 Jena, Germany; 4 Institute of Physical Chemistry and Abbe Center of Photonics (IPC), Friedrich-Schiller-University, Helmholtzweg 4, 07743 Jena, Germany; 5 Institute of Computer Science, Faculty of Mathematics, Physics & Computer Science, University Bayreuth, Universitätsstraße 30, 95447 Bayreuth, Germany

**Keywords:** Multimodal image registration, Open-software, Digital pathology

## Abstract

**Background:**

Multimodal histology image registration is a process that transforms into a common coordinate system two or more images obtained from different microscopy modalities. The combination of information from various modalities can contribute to a comprehensive understanding of tissue specimens, aiding in more accurate diagnoses, and improved research insights. Multimodal image registration in histology samples presents a significant challenge due to the inherent differences in characteristics and the need for tailored optimization algorithms for each modality.

**Results:**

We developed MMIR a cloud-based system for multimodal histological image registration, which consists of three main modules: a project manager, an algorithm manager, and an image visualization system.

**Conclusion:**

Our software solution aims to simplify image registration tasks with a user-friendly approach. It facilitates effective algorithm management, responsive web interfaces, supports multi-resolution images, and facilitates batch image registration. Moreover, its adaptable architecture allows for the integration of custom algorithms, ensuring that it aligns with the specific requirements of each modality combination. Beyond image registration, our software enables the conversion of segmented annotations from one modality to another.

## Background

For several years, brightfield microscopy images of Hematoxylin and Eosin (H&E) staining have served as the gold standard for examining histology samples. While new microscopic imaging modalities have emerged to aid in this analysis, most pathologists still rely on analyzing and labeling brightfield images. When utilizing these annotations in alternative modalities, it becomes necessary to perform image registration, which involves converting multiple images captured from the same scene into a unified coordinate system. These images may originate from different timeframes, perspectives, or devices [1].

Histology image registration is a process that involves aligning microscopy images for various purposes. It is used to combine data from different stain methods [2], create 3D reconstructions from 2D images [3], and perform multimodal registration [4].

Multimodal image registration involves aligning images of the same scene that were captured using different technologies or methods, enabling the integration of information from multiple sources and modalities such as brightfield, fluorescence, or confocal microscopy. This integration enables the creation of a more comprehensive and precise representation of the sample.

The registration of multimodal images in histology is a complex task as it involves addressing various challenges, including large image sizes, repetitive texture, non-linear elastic deformation, occlusions, missing sections, non-rigid deformation, contrast differences, appearance variations, and local structural disparities between slices. These challenges hinder the identification of unique landmarks for alignment, thus affecting the accuracy of registration [2, 5]. It is important to highlight that the utilization of multimodal registration is needed for training data on photonic measurement techniques where the annotations are derived from the standard Hematoxylin and Eosin technique [6].

Multimodal image registration algorithms in histology are designed to optimize registration for specific modalities, which means that not all algorithms are equally effective in registering all types of images. Therefore, careful consideration should be given to selecting algorithms that are well-suited for the specific imaging modalities used. The objective of this work is to develop a cloud-based system for multimodal histological image registration that provides user-friendly interfaces, efficient algorithm management, and comprehensive visualization tools.

### Related work

In recent times, there has been remarkable progress in the development of computer tools for histological image registration. ImageJ, widely regarded as the most popular software for biological image analysis, offers various plugins for image registration, including StackReg, TurboReg, MultiStackReg, Linear Stack Alignment with SIFT, TrakEM2, and BunwarpJ [7, 8]. Each of these plugins employs distinct methods for image registration, and ImageJ can be utilized as a viewer to compare the results.

QuPath is another open software for bioimage analysis, that provides an extension specifically designed for histological image registration and alignment [9]. However, it lacks the flexibility to incorporate new algorithms, which means that if the registration results are unsatisfactory, manual registration becomes necessary for users.

Wsireg, a recently released open-source software developed by Patterson & Manz, enables multi-modal or mono-modal whole slide image registration using a graph structure [10]. This software harnesses image registration algorithms offered by Elastix [11]. Notably, Wsireg lacks a graphical user interface and requires programming skills for parameter modification.

A semi-automatic workflow for histological image registration was introduced in 2022 by N. Chiaruttini et al., demonstrating the registration of fluorescent images with immunohistochemistry images [12]. This workflow incorporates Fiji, QuPath, and Elastix as integral components. The project continues to evolve under the name “Warpy” [12, 13].

Based on the above-summarized state of the art, it is clear that there is a requirement for a more user-friendly, adaptable, and accessible tool that enables users to explore a broad range of registration methods while avoiding the intricacies associated with existing solutions. Additionally, to our knowledge, no existing software possesses the capability to transfer annotations across different modalities. This feature could substantially enrich the multimodal analysis of histological images, presenting an unexplored avenue in current software offerings.

In response to the demand for technical solutions facilitating the exploration of various methods for multimodal histological image registration, we have created an open-source cloud-based system. This system offers diverse visualization tools, a project manager, and an algorithm manager using a plugin architecture. Notably, our software boasts a user-friendly graphical interface that eliminates the need for users to modify registration parameters. Moreover, algorithm developers have complete freedom to add new tools. The software’s distinctive features include the ability to register segmented annotations alongside images, a simple visualization interface independent of external software like QuPath or ImageJ, cloud collaboration capabilities, and automatic transformation of a pyramidal organization across all images.

## Implementation

The development of the MMIR was done with the Django framework v4.1, as back-end, and Javascript, CSS and HTML5 for the front-end. Bootstrap 5.2 was also used to ensure a responsive design that adapts to different screen resolutions. To facilitate its deployment or its use in other computer systems, the system is fully dockerized. MMIR can be put into production on a server or locally using two containers, one with the MySQL relational database and a second container with the web application.

The choice of a cloud-based and dockerized system offers several advantages for our diverse user group. Cloud deployment ensures scalability and accessibility, allowing users to access the MMIR from various locations without hardware constraints. Dockerization simplifies deployment across different environments, making it easier to maintain consistency and portability. This setup significantly reduces the setup overhead for users, enabling quicker onboarding and utilization.

**Fig. 1:**
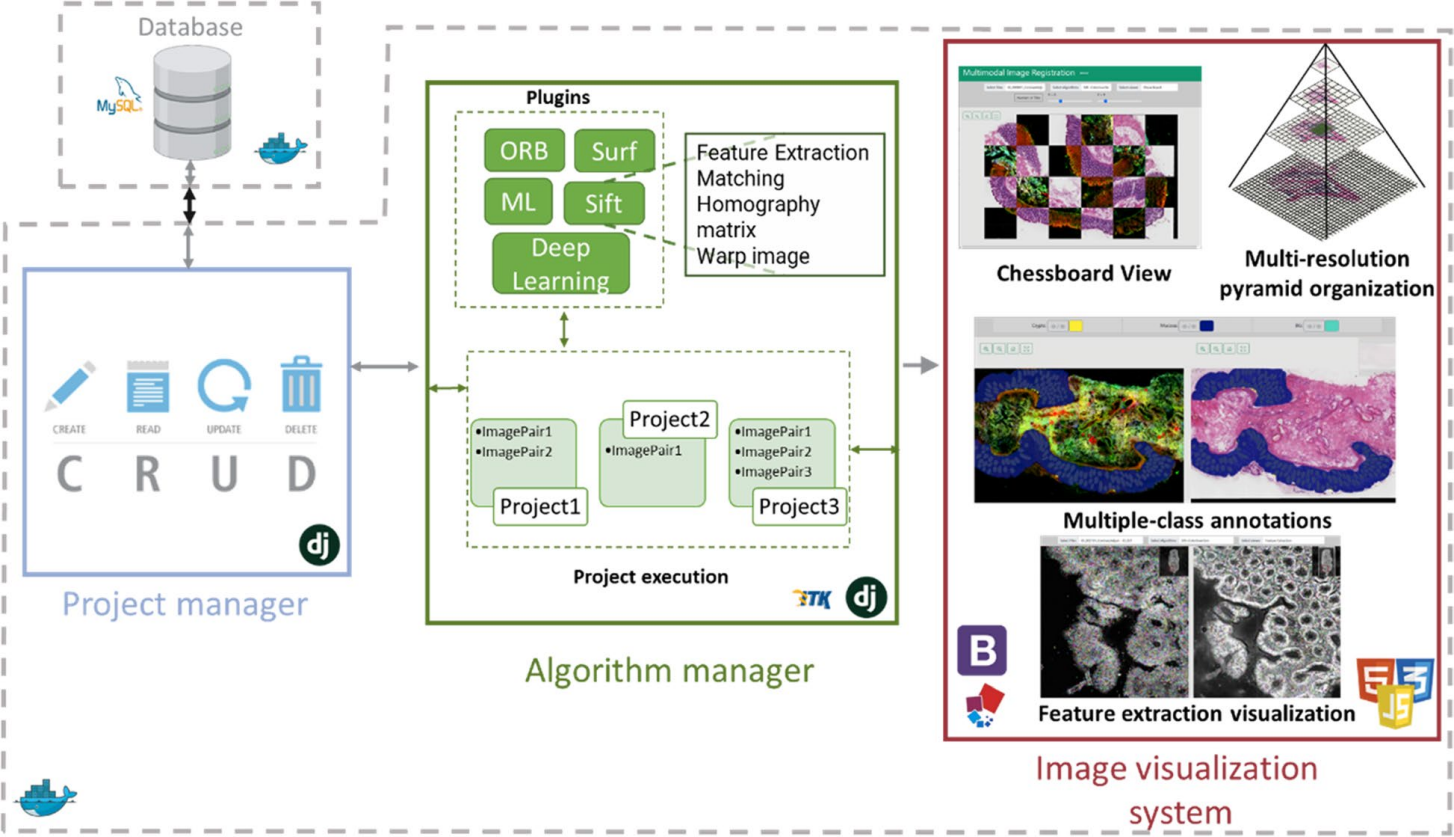
MMIR software architecture, with three core components. A project manager, an algorithm manager, and an image visualization system

The software architecture is organized into three main elements (Fig. 1): a project manager, an algorithm manager, and an image visualization system. Each of these elements brings different features that make our solution unique.

A typical workflow for a user involves the following steps:

1. Creation of a new project, which includes specifying the project name, selecting the images to be registered, choosing the image registration algorithms (from the plugin list) to be used, and loading the segmented annotations associated with the images. During this phase, the project manager is responsible for saving all project-related information in the database and creating a multi-resolution pyramid file for each image in the project.
2. Requesting the execution of all algorithms associated with the project. At this step, the algorithm manager calls and executes the algorithms associated with the project and saves the results in the corresponding folders.
3. Viewing the registration results. In this stage, the image visualization system is in charge of showing the pyramid multiresolution image corresponding to the view selected by the user.
4. Downloading the image registration results.

### Project manager

The project manager provides the necessary features for managing each project. It allows for establishing the connection with the database as well as performing the necessary operations to create, read, update, and delete a project.

#### Segmented annotations

**Fig. 2:**
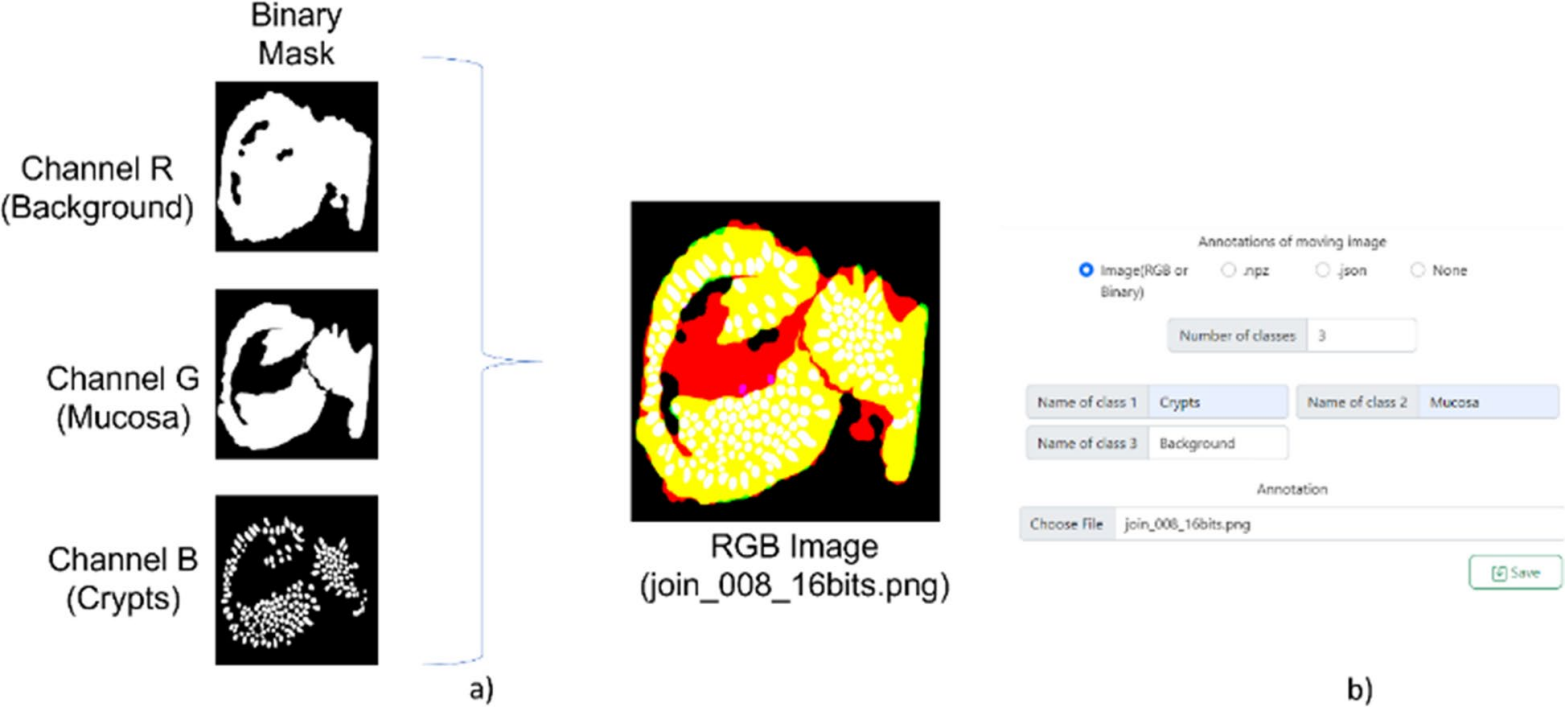
Example of segmented annotations using images. **a** Image Annotation Format: Each channel of the image corresponds to a distinct class annotation. If only a single class annotation is required, a single binary mask can be loaded. **b** Corresponding Filled-in Example

The MMIR system offers users the capability to load segmented annotations for moving images and align them with the coordinate system of fixed images. This functionality enables users to apply annotations created in one modality to a completely different modality, facilitating the transfer and utilization of annotations across various modalities. There are three different methods for uploading segmented annotations: image files (RGB or binary image), npz files, or JSON files with the COCO format. The MMIR system supports loading 8-bit and 16-bit images, both with 3-channel and 1-channel variations. The supported image formats are the same as those handled by the *imread()* function in the openCV library [14]. When RGB images are used, each channel corresponds to a different class and must have a binary mask. Figure 2 provides an example of an RGB segmented annotation depicting three distinct classes: *Crypts*, *Mucosa*, and *Background*. Since the images are read with openCV, the parameters should be filled in the BGR order, as illustrated in Fig. 2b. In the case of a binary image, it will only contain a single class.

**Fig. 3:**
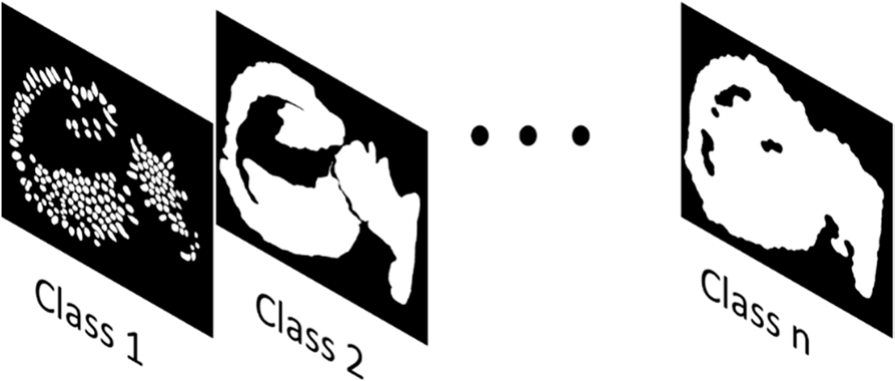
Adaptable multichannel array format for multiple segmented annotation classes. MMIR allows the load of n number of classes using an array, the required array format is [x, y, nC], where ‘x’ and ‘y’ correspond to the images dimensions, and ‘nC’ denotes the number of classes

In scenarios where there are more than three classes, a multidimensional array can be created and saved as an npz file. The array follows the same format as the image annotations format, providing the flexibility to incorporate any desired number of channels, as shown in Fig. 3. The required array format is [*x, y, nC*], where ‘*x*’ and ‘*y*’ correspond to the images dimensions, and ‘*nC*’ denotes the number of classes.

MMIR also accepts JSON files that follow the COCO format [15]. The COCO format is widely used for datasets that focus on object segmentation. It provides comprehensive information in a structured way to describe and label objects in images or videos. In simpler terms, it is a way to define each labeled object by its category (e.g., “cat” or “car”), its location in the image (with a bounding box), and additional information like segmentation masks (to outline the object’s shape) and key points (for detailed object landmarks). This format is helpful for researchers and developers to create and share datasets for training and testing machine learning models that can recognize and understand objects in visual data.

**Fig. 4:**
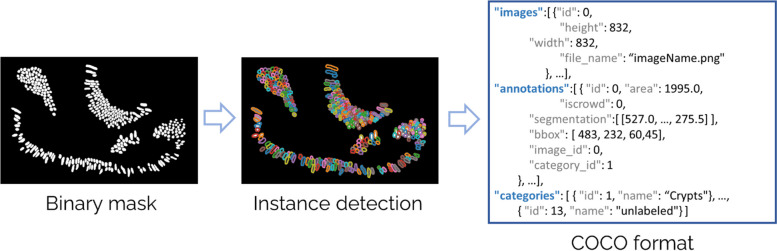
Process to convert a npz file or an image annotation to the COCO format. This figure provides a step-by-step visual guide to the conversion process. It begins with channel extraction, proceeds to instance detection and polygon assignment for each instance, and culminates in the translation into the COCO format

Regardless of the chosen annotation method, all segmented annotations will be converted to the COCO format and stored as JSON files. When a new project is created with image annotations or .npz files, a function is invoked to detect the contours within each binary mask. This information is then leveraged to transform the image into the COCO format, as depicted in Fig. 4.

In MMIR, all segmented annotations are converted into instance segmentation. This means that the system identifies the class of each pixel and distinguishes individual instances or objects within that class. To achieve this, the system first searches for contours inside the binary mask using the marching squares method. Then, a function is called that closes any open contours and extracts the polygon from each contour. Finally, the polygon is saved in COCO format, using the coordinated system of the binary mask.

### Algorithm manager

**Fig. 5:**
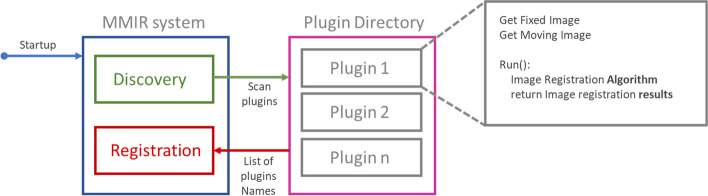
Plugin life cycle. The process begins with system initiation and confirmation of a successful database connection. Subsequently, the algorithm manager initiates plugin discovery, scanning all the plugins within the plugin directory. Finally, all the plugins found are registered. After the successful registration of plugins, they become accessible for utilization in any project

MMIR employs a plugin architecture to incorporate additional algorithms. The plugin life cycle can be seen in Fig. 5. Upon initiating the system and confirming the successful connection to the database, the algorithm manager is activated. It ensures that all plugins within the designated folder are integrated into the system. Once the plugins are successfully registered, they become accessible for utilization in any project.

In addition to managing the life cycle of the plugins, the algorithm manager manages the execution of each project. Each project has one or more image pairs, and for each pair, all the algorithms associated with that project must be executed. In the MMIR system, plugins can only be added for image registration purposes and cannot be employed to expand other functionalities.

Each plugin receives a fixed image and a moving image (in raw resolution) as input and must return a dictionary with eight possible keys: *succ*, *warping*, *Homography*, *f_mov*, *f_fix*, *line_matching, metrics, and messages*. Among these keys, *warping* and *succ* are mandatory for all plugins.

The *warping* key contains the transformed moving image within the updated coordinate system after the registration is carried out. The *succ* key is a boolean value that serves as a flag to determine if the algorithm was executed correctly. If the value is False, the user interface will display an error during the algorithm execution. Additionally, the key *messages* allow the transmission of error messages, providing users with insights into the cause of the error.

The *Homography* key is only mandatory when the project includes annotations. It holds the homography matrix, a 3 × 3 matrix used to transform points between different planes. Most image registration methods employ this matrix to generate the warped image. In MMIR, the homography matrix is utilized to transform each annotation to the new coordinate system. All annotations are converted into the COCO format, resulting in one polygon per class instance. These polygons are then transformed using the homography matrix.

The remaining dictionary elements are optional. *f_mov* represents an image displaying the features detected in the moving image, while *f_fix* shows the features detected in the fixed image. *line_matching* is an image illustrating the matching points between the two images using lines. Lastly, *metrics* provide optional metrics for measuring the registration performance. Although a visual evaluation of the registration is often sufficient, this element allows for the possibility of incorporating evaluation methods within the plugin, such as relative Target Registration Error (TRE), Sum of Squared Differences (SSD), Cross-correlation (CC), Mutual Information (MI), and more. If any of these optional keys are unused, they may return a ‘None value’ in the dictionary.

The design of the algorithm manager enables developers to easily apply various algorithms to different types of modalities. Our tests, detailed in the results section, demonstrate their effectiveness in image registration of brightfield microscopy images with H&E staining, Non-linear Optical (NLO) multimodal microscopy images, and everyday digital camera images. The plugin architecture is versatile, supporting a wide range of registration methods, including rigid and non-rigid techniques, as well as artificial intelligence-based or general-purpose algorithms, among others.

For those interested in creating their own plugins, a plugin template is provided in the repository’s README (https://github.com/BMDSoftware/MMIR). The main/plugins/ folder also includes some plugin examples.

### Image visualization system

**Fig. 6:**
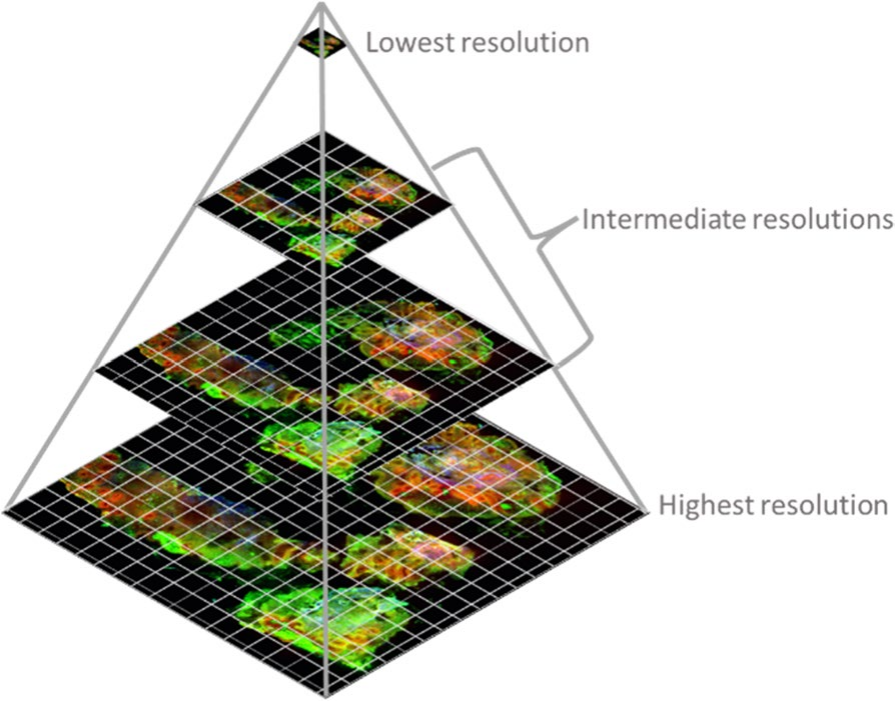
Pyramid image organization. Organization used in all the images that are loaded in MMIR, in this images only is represented 4 levels but an image could have more or less levels depending of the resolution

A single Whole Slide Image (WSI) digitized at a 40x magnification can contain 10^10^ pixels [16]. However, displaying such an image in its original form would require a significant amount of memory. To address this, the image is transformed into a pyramidal organization. In this organization, the image is divided into small fragments called tiles, and multiple levels are created. Each level contains the same image but at different resolutions [17]. The top of the pyramid represents the lowest resolution, while the base represents the original resolution (see Fig. 6).

By organizing the image in this manner, we can load only the necessary tiles into memory for the current viewing space, with the resolution adjusted according to the pyramid level [18, 19]. This allows MMIR to access different levels of the image based on the desired degree of magnification. To generate the multi-resolution images, we utilize the VIPS library [20] (pyvips, in its Python version). By default, MMIR uses tiles with a resolution of 1024 × 1024, and each level of the pyramid is generated by reducing half the resolution of the previous level until reaches a resolution of 1 × 1. The front-end implementation uses the OpenSeadragon library [21] for image visualization. Whether they are input or result images, all images are converted into the pyramidal organization. The conversion process takes place in the backend.

**Fig. 7:**
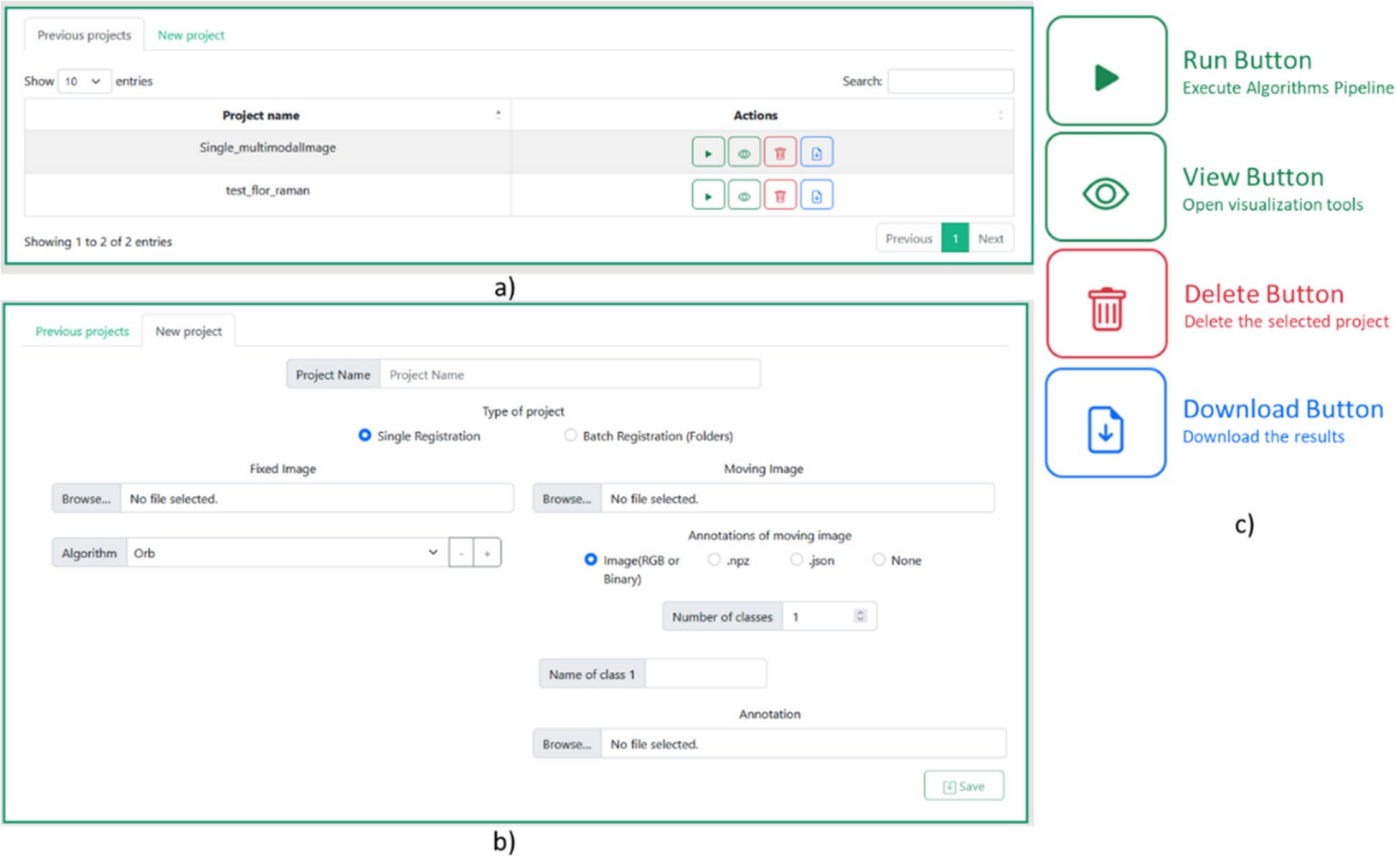
Projects interface. **a** Dynamic table with previous projects, **b** Form for creating a new project, **c** Project action buttons

The user interface consists of two main tabs: *Previous projects* and *New Project* (Fig. 7). Figure 7a shows the mandatory fields for creating a new project. The user needs to provide specific details, such as a unique project name, the images to be registered, the choice of registration algorithms, segmented annotations associated with the images, and the project type. The project type can be either single pair image registration or a batch of images to be registered.

In the case of a batch type project, the user has the option to upload a folder for fixed images and a folder for moving images. It is important to note that the size of both folders must be exactly the same. For example, if there is a need to register five different pairs of images, the user must upload one folder containing five fixed images and another folder containing five moving images. The project manager will automatically sort and pair the images within each folder based on their file names.

Additionally, it is worth noting that different algorithms can be associated with the project through the algorithm selector (Fig. 7b), providing flexibility to evaluate various algorithms simultaneously within a single execution.

Only the paths to the images are stored in the database. The original size images and their multi-resolution representation are stored using the file system storage of Django, inside the media folder.

Once the project is created, it will be available on the main page, where there is a dynamic table with all the existing projects and the different actions that can be performed in each project (Fig. 7b). In MMIR current version, four different actions are provided (Fig. 7c): Execute algorithms (Run button), Open visualization tools (view Button), Delete Project (Delete Button), and download the results (Download Button).

**Fig. 8:**
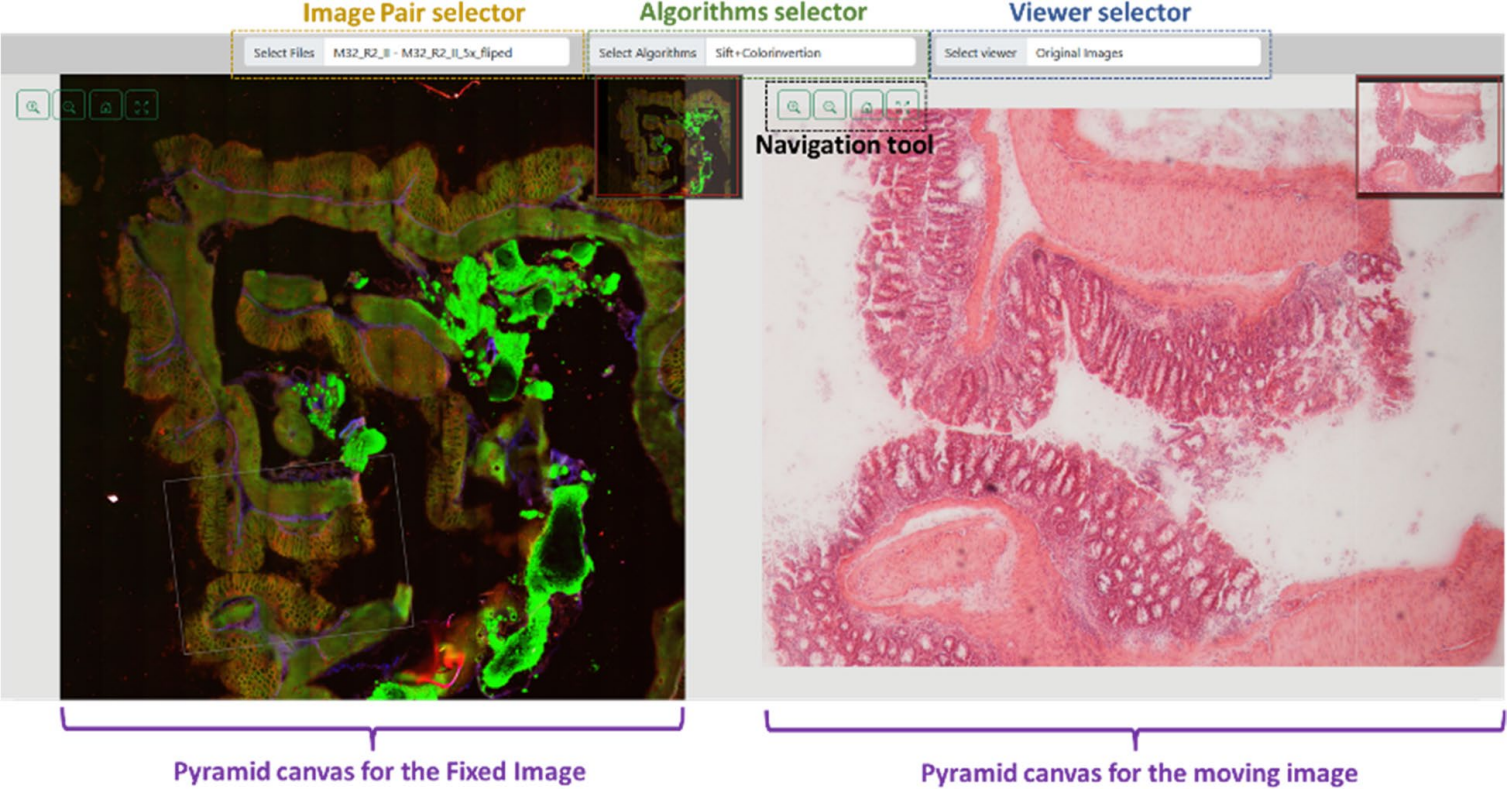
MMIR visualization interface. Three essential selectors can be used, from left to right, image pair selector: explore different image pairs linked to the project, algorithm selector: toggle between various algorithms linked to the project, and viewer selector: access diverse visualization modes

The view button gives access to a new web page with three main selectors (Fig. 8). The first, from left to right, allows the user to view the different image pairs associated with the project. The second selector enables switching between the various algorithms associated with the project. Lastly, the third selector allows access to different types of visualizations.

**Fig. 9:**
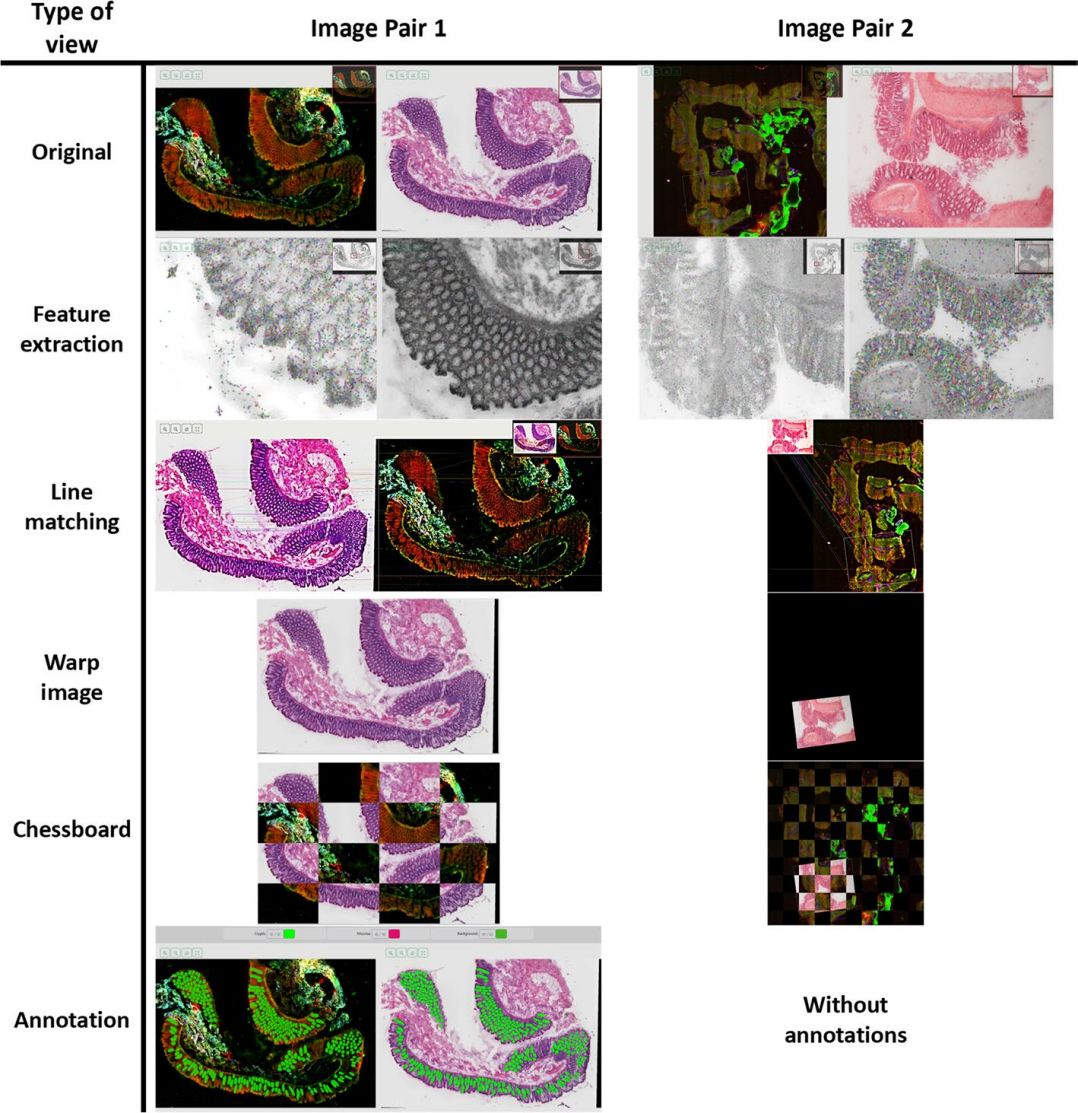
Two pair images examples using different view modes after applying an image registration method. Within MMIR, six different view types are available: (1) Original images view, (2) Feature extraction view, (3) Line matching view, (4) Warp image view, (5) Chessboard view, and (6) Annotation view. The availability of these windows depends on the results of each algorithm assigned to the project, as well as whether the project includes annotations or not

Within MMIR, six different view types are available (Fig. 9): (1) Original images view, (2) Feature extraction view, (3) Line matching view, (4) Warp image view, (5) Chessboard view, and (6) Annotation view. The availability of these windows depends on the results of each algorithm assigned to the project, as well as whether the project includes annotations or not.

When creating a new project, only the “Original images view” option is available. If annotations are added, the “Annotation view” option becomes accessible. Once the user executes all the algorithms associated with the project, the other view options are enabled based on the results of each algorithm. The basic structure of the plugins dictates that a dictionary containing result information must be returned, i.e., if an algorithm does not return a result associated with some of the views, those views will not be available.

## Results

MMIR is a versatile tool for registering high-resolution images of various types. While initially designed for histological imaging, it can be applied to register other image types as well, thanks to its flexibility achieved through the integration of new algorithms via plugins. Each plugin can be developed for image registration in different domains.

MMIR was tested in two distinct scenarios. Firstly, in the registration of Non-linear Optical (NLO) multimodal microscopy images and Brightfield microscopy images with H&E staining. Secondly, exploring its use in the registration of non-histological images.

**Fig. 10:**
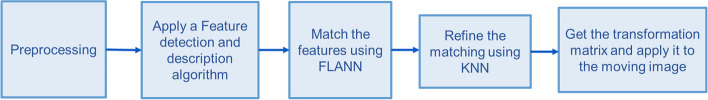
Summarized methodology used in the experiments

For both cases, it was employed a consistent methodology for rigid image registration (see Fig. 10). However, it was adapted the preprocessing steps within each plugin to suit the specific characteristics of the image type. In H&E images, e.g., it was applied intensity adjustments considering that the highest intensity in NLOs is in the tissue, whereas in H&E images, it comes from the background.

Two plugins were created. Using ORB (Oriented FAST and Rotated BRIEF) and SIFT (Scale-Invariant Feature Transform) algorithms for feature detection and description. For feature matching, it was utilized FLANN (Fast Library for Approximate Nearest Neighbors). While FLANN generally provides satisfactory results, it may not always be completely accurate. Therefore, it was further refined the matching outcomes using the K-Nearest Neighbors (KNN) approach.

**Fig. 11:**
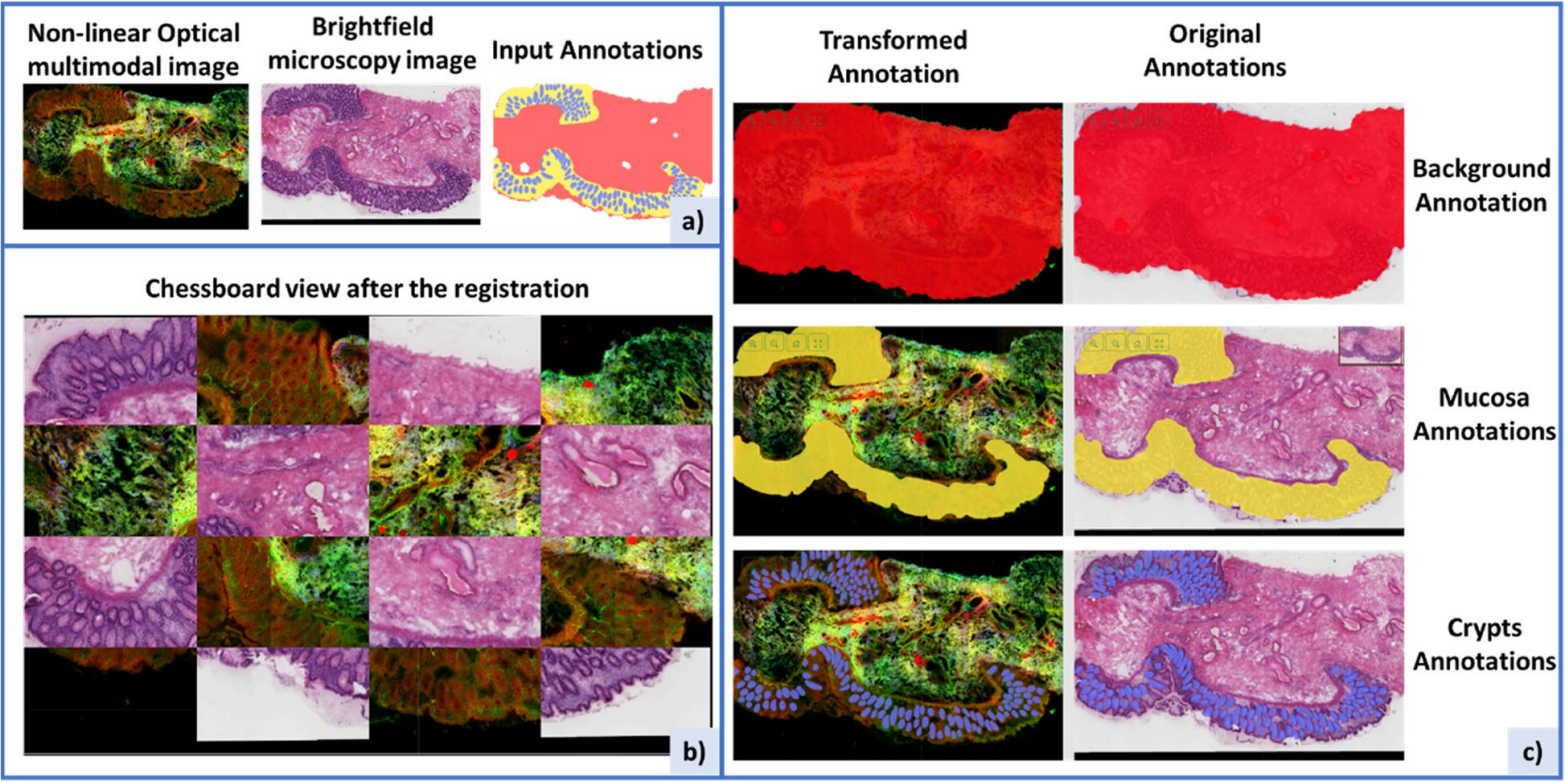
Use of MMIR software in the registration of Non-Linear Optical (NLO) multimodal microscopy images and brightfield microscopy images with H&E staining. **a** Initial inputs for registration, with the NLO image employed as the reference (fixed) image, and the H&E image as the target (moving) image. **b** Visualization of a 4 × 4 chessboard pattern generated post-registration through the utilization of the SIFT algorithm. **c** Demonstration of annotation transformation accomplished via the application of the homography matrix obtained from the image registration process

Due to the complexity of the first scenario, none of the algorithms used works on all samples. However, in specific cases, a good registration has been made. An example can be observed in Fig. 11. In this paper, we do not aim to delve into the intricacies of registering such image types or present a specific algorithm tailored for these domains. These aspects are currently being explored by other members of our team, and their findings will be shared in due course. However, we emphasize that our software plays a main role in evaluating the performance of these algorithms.

**Fig. 12:**
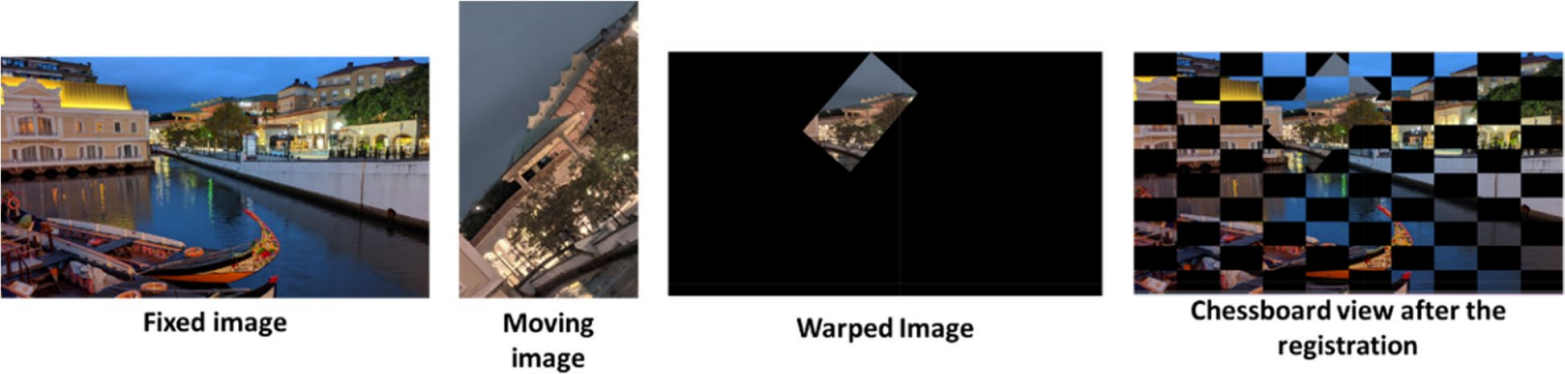
Use of MMIR software in the registration of non-histological images

Furthermore, besides assessing histological images, it was tested the effectiveness of our software with conventional images, which are considerably simpler to register. As expected, our software proves capable of accurately registering these images, even with basic algorithms. Figure 12 demonstrates an example of a successful registration using SIFT.

## Conclusion and future work

In this paper, we have presented MMIR, a cloud-based system for multimodal histological image registration. MMIR addresses the challenges faced in image registration from different modalities by providing efficient algorithm management, user-friendly interfaces, and comprehensive visualization tools, turning it a valuable tool for researchers and pathologists working with multimodal histological images. The system employs a plugin architecture that allows for easy integration of new registration algorithms. The software architecture consists of a project manager, an algorithm manager, and an image visualization system. The project manager handles project management tasks, while the algorithm manager ensures the integration and execution of registered algorithms. The image visualization system utilizes a pyramidal organization to efficiently display very high-resolution images without overload the memory of the users. MMIR supports multiple annotation formats and offers flexibility in loading annotations using different methods.

We have now laid the groundwork for future development and integration of algorithms specifically designed for different histological imaging modalities. Our team is actively engaged in the creation of artificial intelligence based algorithms designed to facilitate the registration of histological images across different modalities. Upon completion of thorough testing, these algorithms will be gradually incorporated into MMIR as new plugins. These advancements will contribute to the creation of a more comprehensive and effective tool for image registration in histology research and clinical applications.

In the future, we aim to enhance the capabilities of our system by enabling the registration of more than two modalities simultaneously. Additionally, we plan to extend the functionality of the system to support three-dimensional reconstruction of histological images. This advancement will provide researchers and clinicians with a powerful tool for examining tissue structures in a more realistic and detailed manner.

## Data Availability

Project name: Multimodal Image Registration (MMIR). Project home page: https://github.com/BMDSoftware/MMIR Operating system(s): Windows or Linux. Programming language: Python, JavaScript, CSS, and HTML. Other requirements: Docker and Docker-compose. License: GNU General Public License v3.0. Any restrictions to use by non-academics: all those set out in the GPL-3.0 license.

## Abbreviations

CC: Cross-correlation.
ER: Entity Relationship
FLANN: Fast Library for Approximate Nearest Neighbors
H&E: Hematoxylin and Eosin
KNN: K-Nearest Neighbors
MI: Mutual Information
NLO: Non-linear Optical
ORB: Oriented FAST and Rotated BRIEF
SIFT: Scale-Invariant Feature Transform
SSD: Sum of Squared Differences
TRE: Target Registration Error
WSI: Whole Slide Image

## References

[1] Zitová B, Flusser J (2003). Image registration methods: a survey. Image Vis Comput.

[2] Borovec J, Kybic J, Arganda-Carreras I, Sorokin DV, Bueno G (2020). ANHIR: automatic non-rigid histological image registration challenge. IEEE Trans Med Imaging.

[3] Pichat J, Iglesias JE, Yousry T, Ourselin S, Modat M (2018). A survey of methods for 3D histology reconstruction. Med Image Anal.

[4] Bulten W, Bándi P, Hoven J, Loo RV, Lotz J, Weiss N (2019). Epithelium segmentation using deep learning in H&E-stained prostate specimens with immunohistochemistry as reference standard. Sci Rep.

[5] McCann MT, Ozolek JA, Castro CA, Parvin B, Kovačević J (2015). Automated histology analysis: opportunities for signal processing. IEEE Signal Process Mag.

[6] Chernavskaia O, Heuke S, Vieth M, Friedrich O, Schürmann S, Atreya R, et al. Beyond endoscopic assessment in inflammatory bowel disease: real-time histology of disease activity by non-linear multimodal imaging. Sci Rep. 2016;6 10.1038/srep29239. 10.1038/srep29239 PMC4942779 27406831

[7] StackReg. http://bigwww.epfl.ch/thevenaz/stackreg/ (Accessed 28 Aug 2023).

[8] ImageJ image Registration. https://imagej.net/imaging/registration (Accessed 28 Aug 2023).

[9] GitHub - qupath/qupath-extension-align: QuPath extension to interactively align images. https://github.com/qupath/qupath-extension-align (Accessed 28 Aug 2023).

[10] Patterson H, Manz T. wsireg: wsireg v0.3.5. 2022. doi:10.5281/ZENODO.6561996.

[11] Klein S, Staring M, Murphy K, Viergever MA, Pluim J (2010). Elastix: a toolbox for intensity-based medical image registration. IEEE Trans Med Imaging.

[12] Chiaruttini N, Burri O, Haub P, Guiet R, Sordet-Dessimoz J, Seitz A (2022). An open-source whole slide image registration workflow at cellular precision using Fiji, QuPath and Elastix. Front Comput Sci.

[13] Warpy. https://imagej.net/plugins/bdv/warpy/warpy (Accessed 2 May 2023).

[14] Bradski G. The OpenCV library. Dr Dobb’s Journal of Software Tools. 2000;

[15] Lin T-Y, Maire M, Belongie S, Bourdev L, Girshick R, Hays J *et al.* Microsoft COCO: Common Objects in Context. 2014. http://arxiv.org/abs/1405.0312.

[16] Gutman DA, Cobb J, Somanna D, Park Y, Wang F, Kurc T (2013). Cancer digital slide archive: an informatics resource to support integrated in silico analysis of TCGA pathology data. J Am Med Inform Assoc.

[17] Marini N, Otálora S, Podareanu D, van Rijthoven M, van der Laak J, Ciompi F, et al. Multi_scale_Tools: a Python library to exploit Multi-Scale whole slide images. Front Comput Sci. 2021:3. 10.3389/fcomp.2021.684521.

[18] Singh R, Chubb L, Pantanowitz L, Parwani A (2011). Standardization in digital pathology: supplement 145 of the DICOM standards. J Pathol Inform.

[19] Hamilton PW, Bankhead P, Wang Y, Hutchinson R, Kieran D, McArt DG (2014). Digital pathology and image analysis in tissue biomarker research. Methods.

[20] Martinez K, Cupitt J (2005). VIPS - a highly tuned image processing software architecture.

[21] OpenSeadragon. https://openseadragon.github.io/ (Accessed 7 Nov 2023).

[22] Bocklitz TW, Salah FS, Vogler N, Heuke S, Chernavskaia O, Schmidt C, et al. Pseudo-HE images derived from CARS/TPEF/SHG multimodal imaging in combination with Raman-spectroscopy as a pathological screening tool. BMC Cancer. 2016;16 10.1186/s12885-016-2520-x. 10.1186/s12885-016-2520-x PMC4962450 27460472

